# Role of strain echocardiography in patients with hypertension

**DOI:** 10.1186/s40885-021-00186-y

**Published:** 2022-02-15

**Authors:** Jin Kyung Oh, Jae-Hyeong Park

**Affiliations:** 1 Division of Cardiology in Internal Medicine, Chungnam National University Sejong Hospital, Chungnam National University School of Medicine, Sejong, Republic of Korea; 2 Department of Cardiology in Internal Medicine, Chungnam National University Hospital, Chungnam National University School of Medicine, Daejeon, Republic of Korea

**Keywords:** Hypertension, Echocardiography, Hypertrophy, Left ventricular, Strain echocardiography

## Abstract

Hypertension is a well-recognized risk factor for the development of cardiovascular disease, and the early detection of cardiac changes from hypertension can allow reversing these. Hypertensive heart diseases (HHD) refer to the complex and diverse change of the cardiac structure and function secondary to hypertension. Although conventional echocardiography is the most common imaging modality in detecting HHD, it cannot detect subtle changes of cardiac structure in subclinical states. Because strain echocardiography is another echocardiographic modality can detect subclinical myocardial dysfunction by measuring intrinsic myocardial deformation, it became more and more popular in clinical and research fields. In this review article, we described the basic concept of strain echocardiography and summarized several clinical studies showing its clinical utilities in the detection of HHD.

## Background

**Fig. 1:**
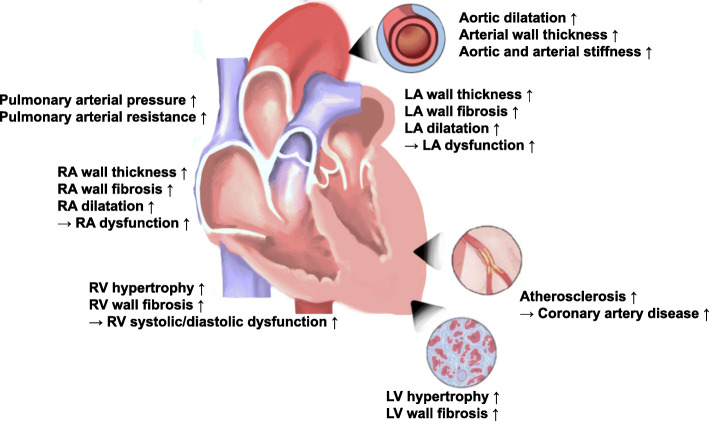
Overview of the structural and functional alterations present in hypertensive heart disease. LA, left atrium; LV, left ventricular; RA, right atrium; RV, right ventricular

Hypertension is the most important cardiovascular risk factor with an increased risk of heart failure (HF), myocardial infarction, stroke, and cardiovascular death [1–4]. Hypertensive heart diseases (HHD, Fig. 1) include cardiac conditions caused by chronically elevated blood pressure, and these include HF, ischemic heart diseases, altered left ventricular (LV) geometry, and LV hypertrophy (LVH) [5–7].

Traditionally, conventional echocardiography is the most common imaging modality in detecting HHD by providing useful structural and hemodynamic findings. The presence of abnormal conventional echocardiographic findings, including abnormal LV geometry and LVH, dilatation of left atrium (LA), and LV systolic and diastolic dysfunction, is associated with poor prognosis associated with hypertension. Speckle-tracking echocardiography (STE) is a non-invasive echocardiographic modality providing myocardial deformation, which helps detect early ischemic changes and myocardial dysfunction, even before symptom onset, undetected by the conventional echocardiographic examination.

In this review article, we described the basic concept of STE and summarized the clinical utility of STE in the evaluation of patients with hypertension.

### Structural and functional changes of the heart in hypertension

Because the target organ damage like HHD from arterial hypertension can be associated with a poor prognosis, identification of these HHD can improve clinical outcomes of these patients. In terms of biochemical alterations, myocardial fibrosis is one of the factors responsible for deterioration of myocardial function in hypertension [8, 9]. Myocardial fibrosis is a major determinant of LVH with the stiff myocardium in HHD, potentially leading to pump failure [10, 11]. Increased myocardial fibrosis and decreased elasticity are characterized by the accumulation of extracellular matrix proteins in the myocardium and surrounding microvasculatures [12]. Cardiac fibrosis alters the myocardial structures, resulting in hypertrophy, chamber stiffness, dilatation of cardiac chambers, conduction abnormalities and arrhythmogenicity, and systolic and diastolic dysfunction of the ventricles and aria [13].

LVH and abnormal LV geometry are common forms of HHD and important independent predictors of adverse long-term prognosis in many cardiovascular diseases. Chronic systolic and diastolic arterial hypertension can cause myocyte hypertrophy. And, hypertension can produce perivascular and myocardial fibrosis, and medial thickening of the intramyocardial coronary arteries [2]. Consequently, hypertension induces global modifications in the cardiac structure and function and is a well-recognized risk factor for developing HF and cardiovascular diseases [14–16]. Because the early detection of LV systolic dysfunction, even in patients without overt symptoms, can give opportunities to reverse their structural changes from hypertension, it is an important issue in hypertensive patients.

LA structural and functional changes can be observed in HHD [17]. Although LA remodeling is closely related to LV structural and functional alterations, it can occur independently from LV remodeling [17, 18]. Also, right ventricular (RV) remodeling can occur due to increased pulmonary arterial pressure. Arterial remodeling is also a common finding in HHD, and hypertension can cause increased stiffening of artery and aortic dilatation, which were associated with an increased risk of cardiovascular diseases [13, 19, 20].

Without prompt antihypertensive treatment, HF and arrhythmias can occur in patients with LVH and abnormal LV geometry. HF with reduced ejection fraction and HF with preserved ejection fraction can be observed in patients with HHD. Ischemic events, including myocardial ischemia and stroke, can also accompany.

### What is myocardial strain and speckle-tracking echocardiography?

Myocardial fibers are a 3-dimensional structure including circumferential fibers in the mid-wall layer and longitudinal fibers in the endocardial and epicardial layers. Myofiber can stretch, shorten and thicken, and these movements in different myocardial layers change myofiber orientation continuously from right-handed helix in subendocardium to left-handed helix in subepicardium [21]. Thus, myocardial contraction occurs clockwise rotation in the midportion and counterclockwise rotation in the apical portion of the LV [22].

Left ventricular ejection fraction (LVEF) as conventional echocardiographic index does not represent intrinsic myocardial property. However, strain echocardiography, a newer echocardiographic modality, can measure myocardial deformation representing the intrinsic myocardial property.

#### Myocardial strain and strain rate

**Fig. 2:**
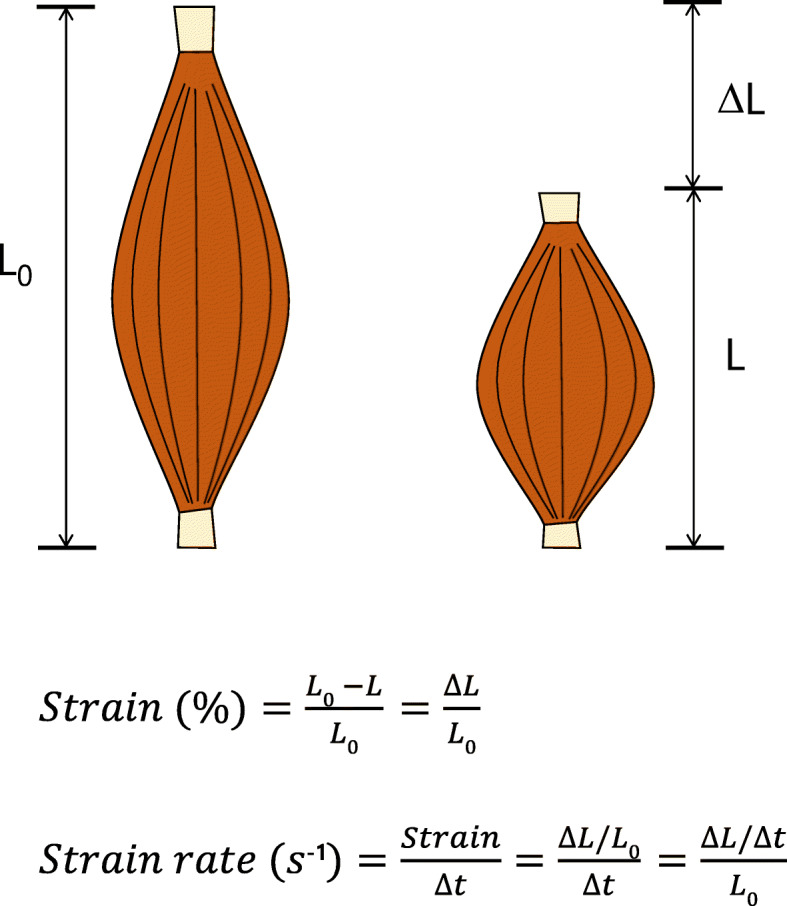
Myocardial strain and strain rate. Simple diagram showing the principle of strain and strain rate. Strain is expressed as a fractional length change, where shortening is a negative value and lengthening a positive value. Strain is calculated as the difference (Δ*L*) of the initial (*L*_*0*_) and the final distance (*L*) between two points divided by the initial distance. Strain rate is the deformation per unit time (Δt), and derives from the ratio between the velocity variation and the initial distance between two points

Myocardial strain is a dimensionless index that can represent myocardial performance. It can be calculated as the change from the original muscle length to the final muscle length after myocardial contraction and presented as a percentage (Fig. 2). During systole, myocardial fibers shorten, and strain value assessing fiber shortening has a negative value while lengthening of fibers during systole, often observed in the segment with dyskinesis, is represented by a positive strain value.

Strain rate considers the time to the strain and refers as a deformation per time unit. The unit of strain rate is s^− 1^ and has the same direction as the strain value, negative value during shortening and positive value during elongation of the myocardium (Fig. 2).

#### Speckle-tracking echocardiography

**Fig. 3:**
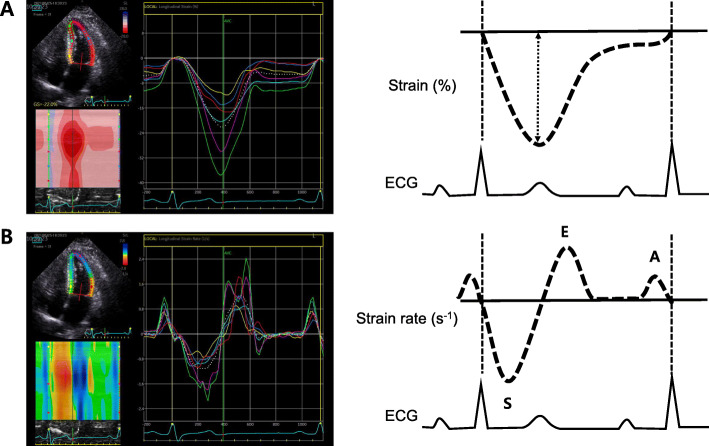
Demonstration of a longitudinal strain and strain rate analysis in the left ventricle using 2-dimensional speckle-tracking echocardiography. Example of a strain curve (**A**) and a strain rate curve (**B**) for one heart cycle

**Fig. 4:**
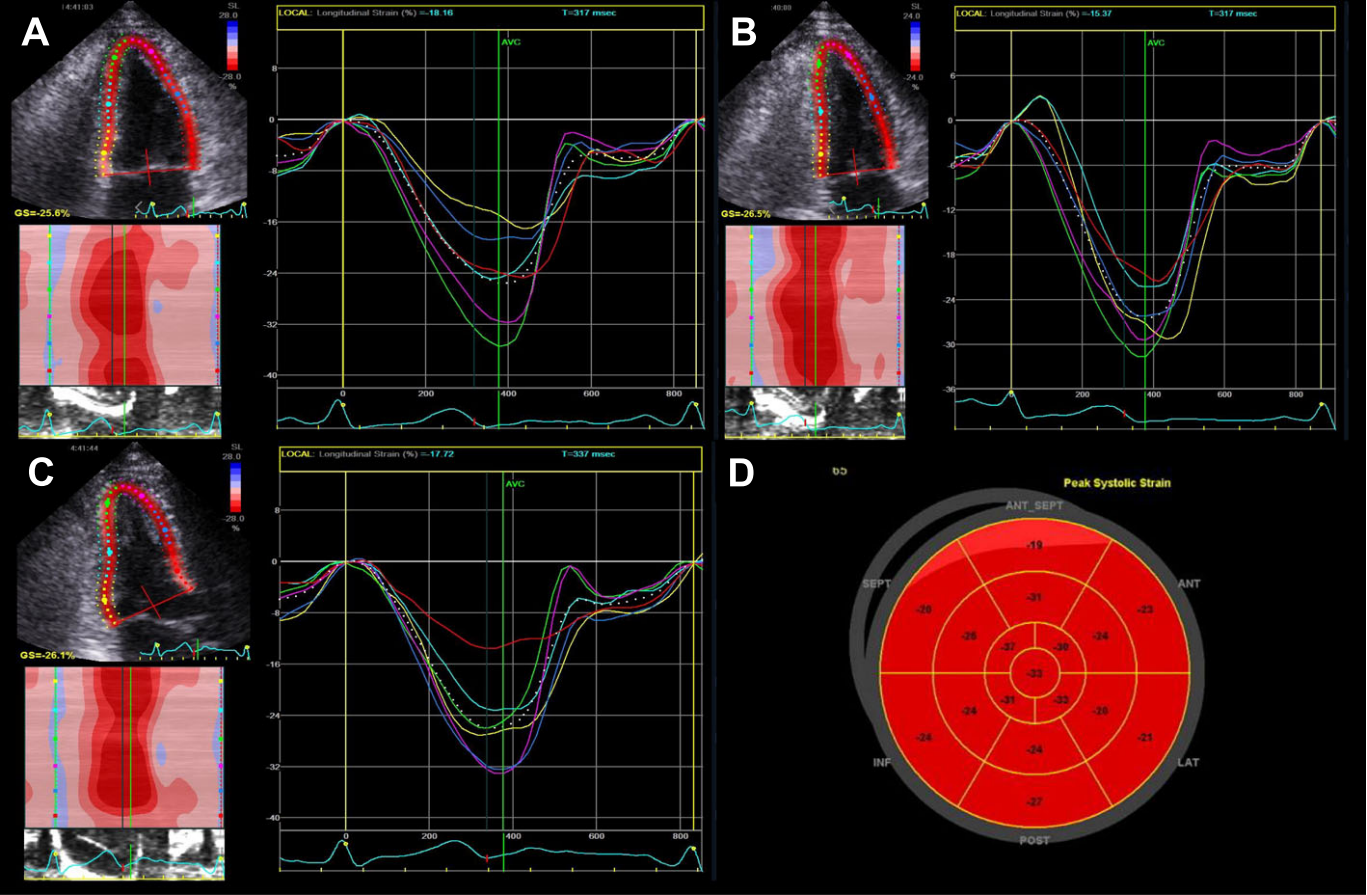
Demonstration of a 2-dimensional strain analysis with GE EchoPAC PC software. After tracing of the endocardial border, the software provides global and regional myocardial strain values automatically in apical 4 chamber (**A**), apical 2 chamber (**B**), and apical 3 chamber views (**C**). The GE EchoPAC algorithm can provide bull’s eye maps of regional longitudinal strain values (**D**)

The myocardial strain and strain rate assessment is a good marker of myocardial function representing intrinsic myocardial performance [23–27]. Myocardial strain can be usually measured by 2-dimensional STE. It tracks ultrasonic speckles, small myocardial footprints in routine 2-dimensional echocardiographic images (Fig. 3). Myocardial speckles are tracked frame-by-frame in one cardiac cycle, and the strain was automatically calculated by measuring the distances between speckles [28]. Among myocardial deformation with three directions, including longitudinal strain (LS), circumferential strain (CS), and radial strain (RS), LS is the most frequently used deformation component from averaging values of 3 apical views in an easy way (Fig. 4).

There are several factors that can affect left ventricular LS (LVLS) include hemodynamic factors, chamber geometry, myocardial tissue characteristics, and synchrony of myocardial contraction.

The increase in LV afterload or increasing heart rate can lead decrease in LVLS [29]. Also, the change of preload can be related to the change of LVLS [30]. Ventricular geometry can affect strain values. The increase of LV wall thickness and dilatation of LV cavity can be associated with the decrease in LS [31, 32]. Regional LS can be reduced in the areas of infiltrative or myocardial storage diseases [33]. Also, fibrotic myocardial areas, usually observed in the myocardial infarction, are associated with a decrease in regional LS. Inhomogeneous myocardial activation, usually associated with the left bundle branch block, is associated with the difference in sequence of myocardial contraction of the ventricular septum and the LV lateral wall. Other factors affecting LS include gender and age [34]. Usually, females have better LVLS values than males, and younger adults have better LS values than older adults.

**Fig. 5:**
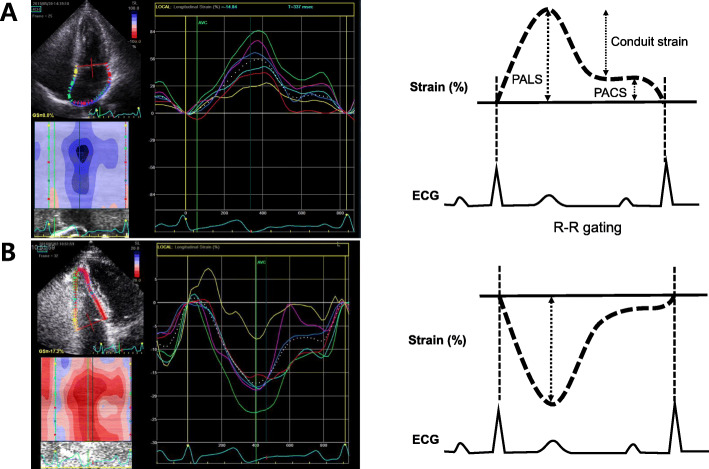
Demonstration of LA and RV strain analysis using 2-dimensional speckle-tracking echocardiography (**A**). LA strain and illustration of the 3 phases of LA function with an R-R gating analysis. RV longitudinal strain calculated as the average of the six-segment model (**B**). ECG, electrocardiography; LA, left atrium; PACS, peak atrial contraction strain; PALS, peak atrial longitudinal strain; RV, right ventricular

**Fig. 6:**
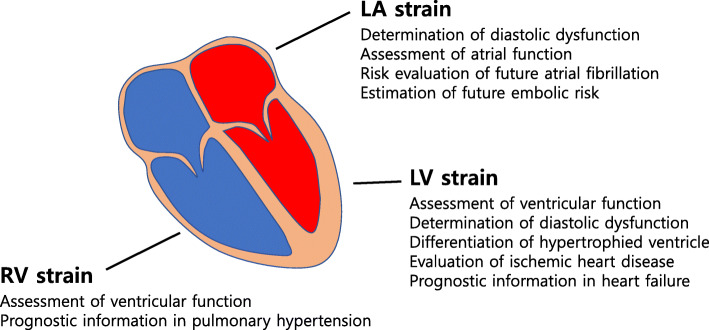
Schematic illustration showing clinical indications of the speckle-tracking echocardiography. Speckle-tracking strain is an increasingly used echocardiographic technology that can provide additional clinical utility. LA, left atrium; LV, left ventricular; RV, right ventricular

STE can measure LA strain (Fig. 5A) and RV strain (Fig. 5B). Thus, STE has become a major echocardiographic modality measuring strain and strain rate in the clinical fields (Fig. 6).

Although echocardiographic strain assessment has relatively high inter-observer and intra-observer variability, as well as inter-vendor variability [35], it should be emphasized that tracking myocardial deformation in systole and diastole provides an additional quantitative measure of myocardial function. Several studies showed that LV global LS represented a better predictor of cardiovascular morbidity and mortality than LVEF in the general population, as well as in HF or various cardiovascular diseases [36–39]. LV multidirectional strain is also associated with LV diastolic function indices (E/e’ and E/A ratios), which means information about LV filling pressure [40, 41]. In addition, strain by STE has grown in importance in both the diagnostic and prognostic evaluation across the cardiac disease spectrum including coronary artery diseases, valvular heart diseases, cardiomyopathies, and RV dysfunction.

### Clinical applications of speckle-tracking echocardiography in patients with hypertension

There are several clinical utilities of STE in patients with arterial hypertension (Fig. 6 and Table 1).

**Table 1:** Summary of clinical studies on STE in patients with hypertension

Reference	Study subject	Method	Parameter	Main findings
**Patients with normal geometry**				
Imbalzano et al. [42] (2011)	51 patients with hypertension (mean age 56.5 ± 14 years, 65% males) and 51 controls	2D-STE	LV longitudinal, circumferential, radial strain and twist	LV systolic longitudinal strain was impaired in hypertension patients, including those without LVH. In the patients with LVH, radial strain was reduced, and circumferential strain and twist were increased
Kang et al. [43] (2008)	56 patients with hypertension (mean age 48 ± 11 years, 61% males) and 20 age-matched controls	2D-STE	LV longitudinal, circumferential, radial strain and strain rate, and basal-to-apical torsion	Longitudinal strain was significantly decreased, and basal-to-apical torsion was increased in patient with hypertension and normal EF. Longitudinal and basal-to-apical torsion independently correlated with the serum TIMP-1 level
**Patients with LVH**				
Mizuguchi et al. [44] (2010)	98 patients with hypertension (25% concentric LVH, 43% eccentric LVH) and 22 age-matched controls	2D-STE	LV longitudinal, circumferential, radial strain and strain rate	The systolic LV myocardial deformation was impaired in all the longitudinal, circumferential, and radial directions in patients with hypertension and concentric LVH. The mean peak systolic circumferential strain was an independent predictor related to LVEF
Saito et al. [39] (2016)	388 patients with hypertension and abnormal LV geometry (31% concentric LVH, 22% eccentric LVH, 47% concentric remodeling)	2D-STE	LVGLS and GCS	GLS and its deterioration (> 16%) are related with MACE in asymptomatic hypertensive heart disease, and was very useful for predicting risk of MACE
Lee et al. [45] (2016)	95 patients with hypertension (mean age 65.5 ± 12.0 years, 60% males)	2D-STE	LVGLS of subendocardium, subepicardium	Longitudinal strain of the subepicardial myocardium (> 17.6%) was the only independent prognostic factor in regularly treated hypertensive patients
**Left ventricular diastolic dysfunction**				
Mu et.al [46]. (2010)	75 patients with hypertension and normal LV geometry (mean age 48 ± 11 years, 61% males) and 50 controls	2D-STE	LV longitudinal, circumferential, radial strain rate, and torsion rate	Reduced longitudinal, circumferential, radial strain rate, increased rotation rate, and extension of untwisting half-time are the sensitive indicators to diagnosis hypertensive patients with early LV diastolic dysfunction
Soufi Taleb Bendiab et al. [40] (2017)	200 patients with hypertension and normal LVEF (mean age 61.7 ± 9.7 years, 68% LVH)	2D-STE	LVGLS	Reduced GLS (> − 17.6%) is associated with long-lasting, uncontrolled hypertension, overweight, diabetes, related metabolic changes, and is more pronounced in patients with LVH
Mizuguchi et al. [47] (2008)	70 patients with normal EF and cardiovascular risk factors and 30 age-matched controls	2D-STE	LV longitudinal, circumferential, radial strain and strain rate, and torsion	The mean peak systolic and early diastolic longitudinal strain and strain rate were lower in the E/A < 1 group. LV myocardial contraction and relaxation were first impaired in the longitudinal direction
**Left atrial function**				
Salas Pacheco et al. [48] (2019)	50 patients with hypertension and 80 healthy volunteers	2D-STE	LA reservoir, contraction, conduit strain, and LVGLS	LA strain of pump and reservoir phases, and LA independent strain were lower in hypertensive patients. LA independent strain only correlated with minimum LA volume, and can identify atrial myocyte contractile dysfunction
**Right ventricular function**				
Pedrinelli et al. [49] (2010)	89 patients with office BP varying from the optimal to mildly hypertensive range	2D-STE	RV longitudinal peak strain and strain rate	RV peak systolic strain and early diastolic strain rate reduced in the mid-tertile of BP distribution. RV systolic and diastolic strain indices correlated inversely with increasing septal thickness.
Tumuklu et al. [50] (2007)	35 patients with hypertension and 30 age-and sex-matched controls	2D-STE	RV longitudinal peak strain and strain rate	RV peak systolic strain was significantly lower in hypertension patients with and without LVH in comparison with normotensive controls
Tadic et al. [51] (2014)	59 untreated hypertension patients, 62 well-controlled hypertension, 58 treated but uncontrolled hypertension patients, and 55 age-and sex-matched controls	2D-STE	RVGLS and strain rate	RVGLS was significantly decreased in untreated and uncontrolled hypertension patients comparing with controls and well controlled participants. RVGLS and 3D RV stroke volume were independently associated with peak oxygen uptake.

*BP* blood pressure, *EF* ejection fraction, *GCS* global circumferential strain, *GLS* Global longitudinal strain, *LA* left atrium, *LV* left ventricle, *LVH* left ventricular hypertrophy, *MACE* major adverse cardiac events, *RV* right ventricle, *RVGLS* right ventricle global longitudinal strain, *STE* Speckle-tracking echocardiography

#### Hypertension patients with normal left ventricular geometry

It is well known that a preclinical LV systolic dysfunction occurs in hypertensive patients with LVH [44]. Even before LVH occurs, hypertension leads to early changes of LV mechanics. However, the conventional transthoracic echocardiography is usually unable to detect early subtle abnormalities in LV systolic function caused by arterial hypertension, prior to manifestation of LVH. The STE can identify subtle adaptive changes of LV systolic mechanics in hypertensive patients without symptoms or signs of HF and with normal contractile function. Patients with hypertension without LVH show impaired systolic LS compared with healthy control participants (− 18.0% ± 1.9% vs. –20.4% ± 2.5%, *P* = 0.02). In contrast, Doppler tissue imaging (DTI) was able to detect an LV systolic dysfunction only in the patients with LVH (*P <* 0.001) [42]. Moreover, in patients with LVH, STE showed lower LS (− 15.9% ± 3.3% vs. –20.4 ± 2.5%, *P* < 0.001) and RS (40% ± 20% vs. 54.5% ± 16%, *P* = 0.02), higher CS (− 22% ± 4% vs. –20% ± 2.8%, *P* = 0.05), and twist (23.8° ± 5.2° vs. 12.5° ± 4.8°, *P* < 0.001) than controls. Although basal LS by DTI was not evaluated, velocity measures were normal in hypertensive patients without LVH. This finding demonstrates the advantages of 2-dimensional strain over DTI in the early detection of LV systolic dysfunction. Early impairment of myocardial contractility may be secondary to hemodynamic or biochemical changes. The increased end-systolic wall stress plays a crucial role in leading to longitudinal dysfunction in HHD. Chronic increase in end-systolic wall stress promotes the subendocardial synthesis of collagen, reducing longitudinal deformation.

Kang et al. [43] revealed that impaired LS and increased LV torsion are associated with higher serum levels of tissue inhibitor of the metalloproteinase-1 matrix, which controls myocardial collagen turnover, in patients with hypertension and normal LVEF. They demonstrated that excessive accumulation of fibrillar collagen could progress to myocardial fibrosis and contribute to early LV contractile dysfunction. Because arterial hypertension has impact on all myocardial layers, endocardial, midcardial and epicardial LS were lower than controls, and epicardial LS was important predictor for cardiovascular events including cardiovascular mortality and rehospitalization in hypertensive population [45]. On the other hand, RS and CS, as well as twist, were preserved in hypertensive patients without structural LV changes in this study. These findings fit the novel criteria of HF classification, stating that only transmural damage was associated with reduction of LVEF, CS and twist, whereas subendocardial dysfunction results in isolated LS impairment [52].

#### Hypertensive patients with LVH

LVH is a common response to chronically elevated afterload, enabling normalization of LV wall stress and preservation of LV mechanical function [53]. LVH is an independent predictor of cardiovascular morbidity and mortality in hypertensive patients [54, 55]. This structural remodeling is associated with various alterations including myocardial stiffness, impaired vasomotor reactivity of coronary artery, depressed LV wall mechanics, and abnormal LV diastolic filling pattern [56–60].

A previous study indicated that longitudinal LV contractility was decreased in healthy elderly people, and in patients with hypertension, diabetes, hypertrophic cardiomyopathy, or diastolic HF [61–64]. The aging process promotes fibrosis of the subendocardial myocardium in normal subjects, and the connective tissue content increases in hypertensive patients with LVH [65, 66]. Myocardial fibrosis related to pressure overload is frequent in the subendocardial layer, and it is well known that there is a close association between abnormal longitudinal function and content of interstitial fibrosis [67]. Therefore, LV longitudinal function is deteriorated in healthy elderly people and in hypertension patients with or without LVH. In general, radial myocardial thickening is closely related to LV systolic function, and even if longitudinal LV function is deteriorated, radial function is still maintained. A previous study using 2-dimentional strain imaging reported that LV diastolic and systolic function were first impaired in the longitudinal direction in asymptomatic patients with cardiovascular risk factors and preserved LV pump function. In systole, radial thickening is preserved, and as the longitudinal shortening decreases, the circumferential shortening increases for maintenance of LVEF [47]. In addition, Mizuguchi et al. [44] indicated that concentric LVH caused systolic LV myocardial deformation in all 3 directions including longitudinal, circumferential, and radial in hypertensive patients. However, LV pump function were compensated by increasing circumferential shortening at ventricular systole. Thus, increase in circumferential shortening might be major compensatory mechanisms for maintaining LV pump function even when the longitudinal shortening is decreased.

#### Diastolic dysfunction in HHD

Age-related changes in LV relaxation have recently facilitated the detection of impaired LV diastolic function as reflected by E/A < 1 and a prolonged deceleration time of early diastolic transmitral flow with the widespread use of pulsed Doppler echocardiography. Several studies reported that diastolic LV dysfunction precedes systolic LV dysfunction especially in patients with cardiovascular risk factors such as hyperlipidemia, diabetes, hypertension, obesity, and smoking habits [68–72]. LV multidirectional strain is associated with LV diastolic function indices such as E/e’ and E/A ratios that enables information regarding LV filling pressure [40, 41, 73]. In addition, strain rates (early and late diastolic and systolic) represent strain equivalent for tissue Doppler-derived parameters and correspond well with indices of LV diastolic function. In diastole, the mean peak early diastolic longitudinal strain rate decreased in longitudinal and radial directions, particularly in the former direction. There was a positive correlation between the longitudinal early diastolic strain rate and E/A. In addition, the peak LV longitudinal strain rate during atrial systole was an independent predictor related to E/A in all patients [46, 47]. However, there was no significant increase in pressure at the end of the LV diastole because sufficient LV filling was achieved by effective atrial contraction. Therefore, longitudinal LV myocardial deformation is an important marker to detect subclinical changes in LV contraction and relaxation. Because LA structure and function are directly influenced by the LV filling pressure, LA assessment is an essential step in the diagnosis of diastolic dysfunction. LA strain derived from STE can help assess LA function objectively through the 3 distinct phasic motions of the LA cycle, which are significantly influenced by the LV diastolic strain rates and LV global LS.

Previous study demonstrated that hypertension is directly associated with LA volume change, and it has been demonstrated that despite normal LA volume, there is abnormal LALS in hypertension [74]. This shows that the earliest abnormality in hypertension appear as a decrease in LA strain, followed by LA dilatation, subclinical LV dysfunction, and finally HF with normal or reduced LVEF [2]. Salas Pacheco et al. [48] found that in patients with HHD, the indexed LA volume was greater than in the control group (34 ± 7.8 mL/m^2^ vs. 24 ± 4.9 mL/m^2^); strain of pump (− 5.7% ± 2.4% vs. − 17% ± 3.5%) and reservoir phases (34% ± 9% vs. 48% ± 10%) were worst. The minimum LA volume was higher (26 ± 10 mL vs. 15 ± 8 mL) and LA independent strain was lower in hypertensive patients (4.0% vs. 6.5%, *P* = 0.001). The LA independent strain quantifies LA reservoir phase deformation during isovolumetric relaxation. These findings suggest that in the early stages of HHD, the LA experiences a remodeling with dilation and reduction of LS in the pump and reservoir phase while sustains normal LVEF and global LS. LA strain represents a new tool in the evaluation of atrial function. STE is more sensitive method to initial changes in myocardial function and independent of the insonation angle, but since the absolute value is affected by the hemodynamic loading condition, the evaluation of LA strain during isovolumic relaxation may reflects a condition independent of the hemodynamic loading and traction by the atrioventricular plain [75]. In addition, the lower value of independent strain in hypertensive patients shows that there is intrinsic atrial myocyte damage with significant dysfunction in the early stages [48].

#### RV function in HHD

Arterial hypertension can impact pulmonary circulation and change pulmonary arterial structure. Sequentially, these can provoke RV remodeling. Common pathophysiologic mechanisms include common pathways of arterial hypertension and impaired LV diastolic function as results of increased LV afterload. Overstimulation of the sympathetic system and the renin—angiotensin—aldosterone system can alter pulmonary vascular structure and increase pulmonary vascular resistance. Increased pulmonary vascular resistance impairs RV function and causes RV hypertrophy, subsequently [76]. Arterial hypertension induces LV hypertrophy and cause LV diastolic dysfunction. In patients with LV diastolic dysfunction, increased LA pressure can be delivered to pulmonary capillary and pulmonary artery. Prolonged increased LA pressure can induce pulmonary hypertension as results. Increased RV filling pressure, RV hypertrophy and ventricular interdependency can cause RV systolic and diastolic dysfunction in patients with arterial hypertension.

Conventional echocardiographic study is insufficient in the demonstration of RV dysfunction in patients with arterial hypertension in several studies [50, 77–79]. However, strain echocardiography can show subtle changes of RV. In the early stage of RV remodeling, RV longitudinal function is progressively decreased, but transversal function is preserved and even increased due to circumferential fibers of the subepicardial layer. Therefore, compared to the fractional area change remaining within the normal range for a long time and deteriorates last in the cascade, RV LS can be a sensitive parameter of RV function capable of detecting subtle changes at subclinical levels in patients with arterial hypertension. In the first study using speckle-tracking imaging in young hypertensive patients, RV strain and strain rates were deteriorated particularly in apical and mid segments of RV free wall than in the basal RV segment [49]. Tumuklu et al. [50] reported that free wall RV peak systolic strain was significantly lower in both groups of hypertensive patients with and without LVH than in the normotensive controls. The study results of patients with prehypertension revealed that RV global LS was gradually decreased from the subjects with optimal blood pressure, across the prehypertensive subjects to the hypertensive individuals [80]. Tadic et al. [51] showed that untreated hypertensive patients have significantly lower RV global LS compared to well-regulated hypertensive patients and controls, which was similar to poorly controlled hypertensive patients. Furthermore, functional capacity estimated by peak oxygen consumption correlated with RV free wall LS, 3-dimensional RV end-diastolic volume, RV stroke volume, RV ejection fraction, and right atrial LS. However, only RV free wall strain and 3-dimensional RV stroke volume were independently associated with peak oxygen uptake in the study population.

## Conclusion

Hypertension is a well-recognized risk factor for the development of cardiovascular disease. Preclinical LV systolic dysfunction occurs in hypertensive patients and LVH, but even before LVH occurs, hypertension causes early changes in LV dynamics. Therefore, the accurate assessment of their influence on LV systolic and diastolic function in a subclinical state is clinically important for preventing the development of overt cardiovascular diseases. STE can detect subtle changes in myocardial dysfunction that cannot be detected with conventional echocardiography before the overt manifestation of LVH appear.

Both longitudinal LV diastolic and systolic function can be impaired early even in asymptomatic patients with cardiovascular risk factors and preserved LV systolic function. In systolic phase, longitudinal shortening decreases and radial thickening was preserved, but the circumferential shortening increases to maintain the LVEF. In the diastolic phase, the early diastolic strain rate decreased, especially in the longitudinal direction, even if the LV filling pressure did not increase significantly. In this regard, strain appears to be more sensitive than both conventional echocardiography and DTI in identifying a decrease of intrinsic myocardial contractility in hypertensive patients even long before LVH occurs. Thus, the assessment of myocardial contractility by the STE will be useful in the early detection of subclinical systolic dysfunction and change of LV mechanics. Echocardiographic strain provides more insight into subtle changes in LV function in terms of identifying patients at higher risk for HF and clarifying its clinical impact on the prognosis of HHD.

## Data Availability

Not applicable.

## Abbreviations

CS: Circumferential strain
DTI: Doppler tissue imaging
HF: Heart failure
HHD: Hypertensive heart diseases
LA: Left atrium
LS: Longitudinal strain
LV: Left ventricular
LVEF: Left ventricular ejection fraction
LVH: Left ventricular hypertrophy
LVLS: Left ventricular longitudinal strain
RS: Radial strain
RV: Right ventricular
STE: Speckle-tracking echocardiography
